# FastHPOCR: pragmatic, fast, and accurate concept recognition using the human phenotype ontology

**DOI:** 10.1093/bioinformatics/btae406

**Published:** 2024-06-24

**Authors:** Tudor Groza, Dylan Gration, Gareth Baynam, Peter N Robinson

**Affiliations:** Rare Care Centre, Perth Children’s Hospital, Nedlands, WA 6009, Australia; Telethon Kids Institute, Nedlands, WA 6009, Australia; School of Electrical Engineering, Computing and Mathematical Sciences, Curtin University, Bentley, WA 6102, Australia; SingHealth Duke-NUS Institute of Precision Medicine, Singapore 169609, Singapore; Western Australian Register of Developmental Anomalies, King Edward Memorial Hospital, Subiaco, WA 6008, Australia; Rare Care Centre, Perth Children’s Hospital, Nedlands, WA 6009, Australia; Telethon Kids Institute, Nedlands, WA 6009, Australia; Western Australian Register of Developmental Anomalies, King Edward Memorial Hospital, Subiaco, WA 6008, Australia; Faculty of Health and Medical Sciences, University of Western Australia, Crawley, WA 6009, Australia; Berlin Institute of Health at Charité – Universitätsmedizin Berlin, Charitéplatz 1, 10117 Berlin, Germany; The Jackson Laboratory for Genomic Medicine, Farmington, CT 06032, United States

## Abstract

**Motivation:**

Human Phenotype Ontology (HPO)-based phenotype concept recognition (CR) underpins a faster and more effective mechanism to create patient phenotype profiles or to document novel phenotype-centred knowledge statements. While the increasing adoption of large language models (LLMs) for natural language understanding has led to several LLM-based solutions, we argue that their intrinsic resource-intensive nature is not suitable for realistic management of the phenotype CR lifecycle. Consequently, we propose to go back to the basics and adopt a dictionary-based approach that enables both an immediate refresh of the ontological concepts as well as efficient re-analysis of past data.

**Results:**

We developed a dictionary-based approach using a pre-built large collection of clusters of morphologically equivalent tokens—to address lexical variability and a more effective CR step by reducing the entity boundary detection strictly to candidates consisting of tokens belonging to ontology concepts. Our method achieves state-of-the-art results (0.76 F1 on the GSC+ corpus) and a processing efficiency of 10 000 publication abstracts in 5 s.

**Availability and implementation:**

FastHPOCR is available as a Python package installable via pip. The source code is available at https://github.com/tudorgroza/fast_hpo_cr. A Java implementation of FastHPOCR will be made available as part of the Fenominal Java library available at https://github.com/monarch-initiative/fenominal. The up-to-date GCS-2024 corpus is available at https://github.com/tudorgroza/code-for-papers/tree/main/gsc-2024.

## 1 Introduction

The effectiveness of utilizing ontology-encoded knowledge in rare diseases has been consistently demonstrated through data sharing (Taruscio *et al.* 2015, Boycott *et al.* 2022, Jacobsen *et al.* 2022), as well as in clinical variant prioritization and interpretation (Smedley *et al.* 2016, Clark *et al.* 2018, Son *et al.* 2018) over the years. The Human Phenotype Ontology (HPO) (Robinson *et al.* 2008, Köhler *et al.* 2019), maintained by the Monarch Initiative (Shefchek *et al.* 2020), stands out as the most comprehensive resource for computational deep phenotyping and has emerged as the standard for encoding phenotypes in the rare disease domain. It serves this purpose for both defining diseases and profiling patients to assist in genomic diagnostics, comprising a set of over 16 500 terms describing human phenotypic abnormalities.

HPO-based phenotype concept recognition (CR), i.e. automatic extraction of HPO terms from clinical notes or scientific publications, underpins a faster and more effective mechanism to create patient phenotype profiles or to document novel disease—phenotype or gene—phenotype associations. Lately, the increasing adoption of large language models (LLMs) for natural language understanding has led to the development of models and tools that employ LLMs also for biomedical named entity recognition and CR. Examples of such approaches in the phenotype CR domain include PhenoTagger (Luo *et al.* 2021), PhenoBERT (Feng *et al.* 2023), or PhenoBCBERT (Yang *et al.* 2024).

**Figure 1.**
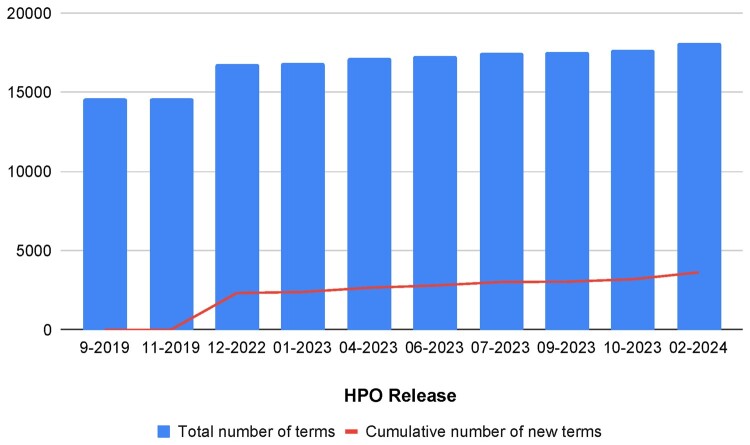
The evolution of the size of HPO in terms of number of concepts between December 2022 and February 2024. The line in the chart represents the growth in new terms being added to the ontology, which for this period was of 1303 terms, i.e. ∼7% increase.

The performance of these models has indeed surpassed traditional dictionary or rule-based approaches (as well as the very few machine learning options—NeuroCR; Arbabi *et al.* 2019), with the underlying publications focusing exclusively on reporting standard efficiency metrics to showcase state-of-the-art results. Unfortunately, given the resource-intensive requirements associated with fine-tuning, we lack an understanding of a real-world deployment of these models—since, to date, no publication has discussed a realistic management of the phenotype CR lifecycle. More concretely, we refer to the following aspects:

- HPO is updated on a monthly basis—and hence the pool of concepts is not only growing but also changing—with terms sometimes subsumed under others or made obsolete—as depicted in Fig. 1.
- The pool of scientific publications—if considering general research or the biomedical domain—grows significantly with every week, at a rate of several hundreds of entries every week.

This leads to two major challenges, currently left untapped:

1. The need to maintain the version of HPO used by the phenotype CR approach up-to-date.
2. The need to efficiently process content to augment previously processed documents (publications, clinical notes, or other corpora) with new terms. Remarkably, the latter is a well-known challenge in the community, and yet has so far been largely ignored. How often would one consider re-processing PubMed, which currently stands at 36 million citations, on a monthly basis (or even the roughly 10% associated with medically relevant abstracts)?

Large language models are resource intensive and, in principle, fairly slow. They could indeed be retrained on a monthly basis to cater for HPO updates. However, both the retraining and the reanalysis would come at a significant cost. It is hence worth considering if the use of the computational resources required to achieve this is justifiable when the same operation can be performed using an insignificant fraction? In this article, we propose a solution that relies on the fundamental pillars of CR in order to cover its two underlying components—boundary detection and entity linking:

- Understanding the domain challenges—in the case of phenotypes, lexical variability.
- Understanding the target ontology—and hence processing the ontology terms appropriately for text mining.

More concretely, we pre-built a large collection of clusters of morphologically equivalent tokens (a total of 573 507 tokens)—to address lexical variability—and have used them to reduce the boundary detection step to spans containing only tokens belonging to ontology concepts.

Our method achieves both state-of-the-art results (0.76 document-level F1 on the standard GSC+ corpus) and a processing speed that enables an effortless deployment in real-world applications by catering appropriately for the CR lifecycle: indexing a new HPO version takes ∼3 min, annotating 10 000 publication abstracts takes ∼5 s (excluding I/O operations). Moreover, additional pre-processing steps can be added to our solution—e.g. extended synonymy—which could rely on existing LLMs or embeddings—without introducing a significant impact on speed.

In addition to proposing a simple yet efficient approach, the contributions of this article also include: a discussion on the challenges associated with evaluating phenotype CR solutions, in general; and an up-to-date version of the GSC corpus.

## 2 Related work

As presented by all other works on the topic, there are two groups of approaches to phenotype CR: machine learning-based methods, in particular the latest approaches using deep learning, and “traditional” dictionary-based methods.

Machine learning-based approaches have dominated the last years in terms of achieving state-of-the-art results, although the availability of gold standard corpora to be used for training is significantly poorer than in other domains. A first such approach was developed by Arbabi *et al.* (2019)—NeuralCR—who employed a neural concept recognizer using a convolutional neural network-based neural dictionary model and tested it successfully on both scientific abstracts and medical notes. PhenoTagger (Luo *et al.* 2021) was the first method to combine dictionary tagging with a BioBERT-based tagger (Lee *et al.* 2019) to efficiently identify HPO concepts—including unseen synonyms and nested subconcepts. PhenoBERT (Feng *et al.* 2023) introduces a two-levels convolutional neural network module—building on NeuralCR’s method, which designed the CNN to take into consideration the hierarchical relationships between HPO terms—before applying BERT. Finally, PhenoBCBERT (Yang *et al.* 2024) is the latest approach in this category and uses Bio+Clinical BERT as its pretrained model.

The second category of approaches—dictionary-based—relies on creating and using inverted indexes from the tokens composing HPO concepts (usually both labels and synonyms), are relatively fast, and provide packaging options that enable deployment in resource-constrained environments, such as typical clinical practices. They achieve high precision at the expense of lower recall rates and tend to struggle with identifying concepts that consist of unseen tokens. The most representative tools in this category are: NCBO Annotator (Jonquet *et al.* 2009), OBO Annotator (Taboada *et al.* 2014), SORTA (Pang *et al.* 2015), Doc2HPO (Liu *et al.* 2019), ClinPhen (Deisseroth *et al.* 2019), and the Monarch Initiative annotator (Shefchek *et al.* 2020). As we discuss in the next section, we propose a solution fitting this category and aimed at addressing the challenges associated with recall. Note that our T-BLAT approach (Groza *et al.* 2023) is complementary to the solution described herewithin since it focuses on typographic errors, however, the current proposal will been implemented as the base CR method in the Fenominal Java package.

## 3 Materials and methods

Our method relies—as any other dictionary method—on three steps, discussed in the following: dictionary creation, indexing of the ontology concepts, and CR (i.e. entity boundary detection + entity linkage).

### 3.1 Dictionary creation

This step aims to address the main challenge associated with phenotype CR: lexical variability. In most cases, lexical representations of phenotypes rely on common English words and hence can take various morphologically equivalent forms—e.g. *short phalanx of the thumb*, *shorter phalanges of the thumbs*, *shortening of the phalanx of the thumb*. This example refers to the same HPO concept and consists of tokens that require morphological consolidation in order to be identified. It is important to note that applying lemmatization in this case will not result in an appropriate consolidation as the lemma of *shorter* is *short*, while the lemma of *shortening* is *shorten*. A similar challenge is encountered for tokens with different localized spellings—e.g. *hypocalcemia—hypocalcaemia* or *haemorrhage—hemorrhage*.

Given the finite set of such lexical variations, we built a comprehensive vocabulary and performed global consolidation of all morphologically equivalent forms. The vocabulary was compiled from all proper words (i.e. tokens formed of letters only) in PubMed abstracts, SNOMED concept definitions, OMIM concept definitions, and GeneReviews pages. A total of 573 507 unique tokens were collected.

The consolidation process consisted of an initial grouping of all tokens sharing the same 4-letter prefix, followed by their re-clustering using OpenAI’s gpt-4.0 model. For each initial group, gpt-4.0 was prompted to “Group the words provided below in a comma separated list between triple ticks into groups denoting the same meaning.” The result was a set of 37 011 clusters of tokens. An example of such a cluster is: [*short, shorter, shorten, shortening,…*]

### 3.2 Ontology indexing

**Figure 2.**
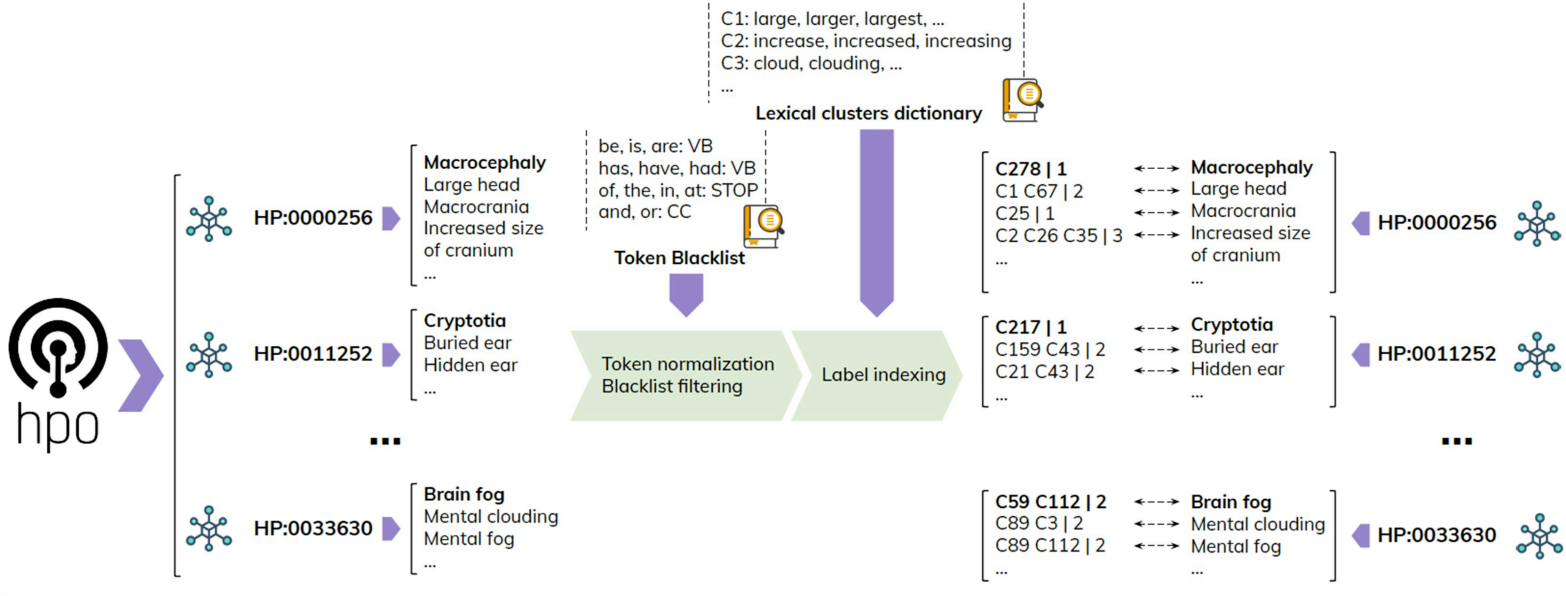
High-level overview of the indexing process. Starting from the left, the labels and synonyms are tokenized, cleaned, and consolidated using the clusters of morphologically equivalent tokens. Sets of such cluster IDs are then serialized as representations of the ontology concepts in the index.

The process of indexing the ontological concepts is depicted at a high level in Fig. 2. For each concept (listed on the left in the figure) we collect the labels and synonyms (labels are represented in bold) and tokenize them—each resulting in a list of words. We then apply a blacklist word-based filter to remove standard stop words (*of, the, in, at, etc.*), verbs (*be, is, are, have, had, etc.*), and conjunctions (*and, or*). The remaining tokens are replaced by the identifier of the cluster they correspond to (note: “C”-prefixed cluster IDs do not have a particular meaning; they are internal to the method, automatically generated when a version of the ontology is indexed and do not represent IDs of concepts from SNOMED or other ontologies). For example the synonym “*Increased size of cranium*” of HP: 0000256 (“*Macrocephaly*”) undergoes the following transformations: tokenization—[“increased,” “size,” “of,” “cranium”], filtering—[“increased,” “size,” “cranium”], consolidation—[C2, C26, C35]. To this last set we include the length of the original label/synonym after blacklist filtering—i.e. [C2, C26, C35–3]—to cater for the rare cases when the same cluster ID is present twice in the label/synonym. The final index consists strictly of associations between ontological concepts and the cluster ID sets representing their label/synonyms (as shown on the right in the figure)—i.e. no other tokens or clusters are retained. Also, note that the original labels and synonyms are retained only to be returned to the user upon CR.

### 3.3 Concept recognition

**Figure 3.**
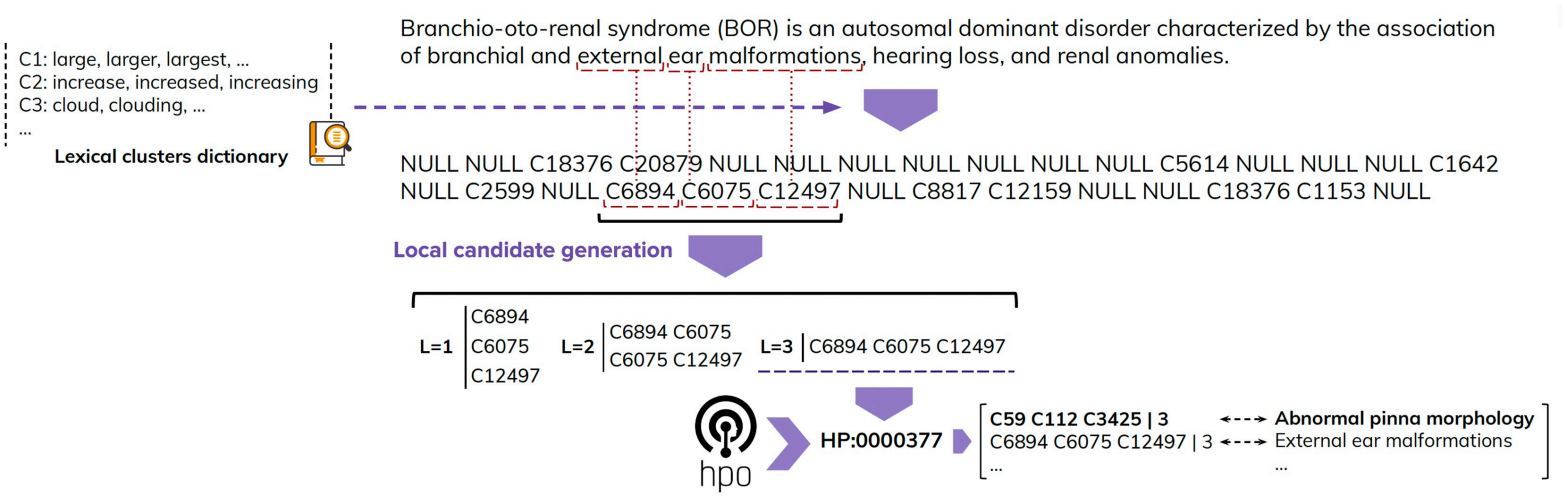
High-level overview of the concept recognition process. The text is tokenized and the tokens are looked up in the ontology index. The gaps left by the tokens absent from the index are used to identify candidates for entity linking.

As opposed to the traditional dictionary-based methods for CR, instead of first looking for candidates in the text by attempting to solve the entity boundary detection problem, we rely on the content of the index and annotate the target text with cluster IDs—as shown in Fig. 3. More concretely, the text is tokenized and each token is looked up in the index (created in the previous step) and, if found, is replaced with the corresponding cluster ID. Note that this operation has an associated complexity of O(*n*), where *n* is the number of tokens in the text. The gaps created by tokens that are not in our index will lead to forming candidates for entity linking. These are decomposed in a combinatorial manner from left to right and from right to left to ensure an appropriate coverage of possibly nested concepts. For example, the candidate [C6894 C6075 C12497] will be decomposed into sub-candidates: C6894, C6075, C12497, [C6894 C6075], [C6075 C12497].

In the final step, all resulting candidates are looked up in the index using the cluster IDs as a set and the total length of the candidate. To optimize the matching of the candidates, each sets of cluster IDs corresponding to labels and synonyms are stored in the index as “signatures,” where a “signature” is a single string concatenating the cluster IDs in a lexicographical order ([C3425, C112, C59] is stored as C112-C3425-C59). Hence a match for the example above [C6894 C6075 C12497] is computed simply by looking up in the index the signature C12497-C6075-C6894. The entire candidate matching operation has an associated complexity of O(*n*), where *n* is the total number of candidates. This not only leads to a linear complexity, but also addresses the challenge of the order of tokens within the label—i.e. *short phalanx* versus *phalanx shortening*.

### 3.4 Experimental setup

#### 3.4.1 Tools

The comparative results discussed in the next section were achieved using the following tools and were reported initially in Groza *et al.* (2023):

- Doc2HPO—via API, with default parameters, during August 10–13, 2022, as per instructions provided at https://github.com/stormliucong/doc2hpo;
- ClinPheno—MacOS download, version available on August 10, 2022 from http://bejerano. stanford.edu/clinphen/. Note that this link is no longer available.
- Monarch Initiative Annotator—via API, with default parameters (match over five characters long), during August 10–13, 2022;
- NCBO Annotator—via API, with default parameters, during August 10–13, 2022;
- PhenoTagger—release v1.1 downloaded from https://github.com/ncbi-nlp/PhenoTagger with models v1.1 downloaded from https://ftp.ncbi.nlm.nih.gov/pub/lu/PhenoTagger/models_v1.1.zip and installed as per the instructions available on August 10, 2022; runs were executed with default parameters.

The results for PhenoBERT and PhenoBCBERT are reported as per the original publications. We will discuss in-depth the challenges associated with comparing these results in the following sections.

#### 3.4.2 Corpora

Two corpora were used to evaluate our solution and to compare the results against those achieved by other tools:

- GSC+: a manually curated dataset that consists of 228 manually annotated abstracts of scientific publications initially annotated and published by Groza *et al.* (2015), and subsequently refined by Lobo *et al.* (2017). For the results initially reported by Groza *et al.* (2015) the annotations were realigned to HPO version 02-2022 by replacing retired HPO IDs with the most up-to-date IDs specified via the alt_id property (operation that left no orphan annotations in the corpus);
- BIOC-GS—the dev component of the corpus made available through Track 3 of BioCreative VIII (454 entries), focusing on extraction and normalization of phenotypes resulting from genetic diseases, based on dysmorphological examination (Weissenbacher *et al.* 2023). An example of an entry is: “ABDOMEN: Small umbilical hernia. Mild distention. Soft.”

GSC+ covers 2773 HPO term mentions, with the minimum size of a document being 138 characters, the maximum size 2417 characters and the average being ∼500 characters. BIOC-GS covers 783 HPO term mentions, with the minimum size of an entry being 13 characters, the maximum 225 characters, and the average ∼56 characters. Note that we chose the dev component of Track 3 because of its similarity in the number of unique HPO IDs to GSC+. We were unable to download the test component of Track 3 and hence the results reported here are not comparable to the results published by the Track’s organizers.

## 4 Results

### 4.1 Performance metrics

Concept recognition, as a task, is conceptually composed of two parts: boundary detection and entity linkage. Boundary detection refers to finding the spans of text that represent candidate-named entities, while entity linkage—given a target ontology—focuses on finding the best concept that matches the candidate named entity. The standard metrics used to report results in the literature are:

- Precision (P)—the ratio between the correctly identified positives (true positives) and all identified positives.
- Recall (R)—the ratio between the predicted true positives and the actual annotation outcomes produced by the tool.
- F1—the harmonic mean of Precision and Recall. F1=2 × P × R/(P+R).

Based on the coverage of the task’s underlying components, the metrics are further refined as:

- Document-level—counting only on the presence/absence of the concept IDs in the given text (publication abstract or clinical note), and hence disregarding the boundary detection aspect, and
- Mention-level—considering each individual mention of the concept in text and its associated position, thereby taking into account boundary detection.

If appropriate for the corpus being tested, mention-level results are reported in addition to document-level results.

### 4.2 Experimental results

To date, most publications proposing a HPO-driven phenotype CR solution report the experimental results using the GSC+ corpus and make reference to a certain version of the ontology. More concretely:

- PhenoTagger—published in 2021, uses the 2019 November HPO release;
- PhenoBERT—published in 2023, uses the 2019 September HPO release—i.e. an earlier version that the one used by the much earlier published PhenoTagger;
- PhenoBCBert—originally published as a pre-print in arXiv in August 2023—does not make reference to an HPO version.

In our previous publication (Groza *et al.* 2023), we used the 2022 February HPO release.

This leads to several challenges. Firstly, each model was trained on a different set of concepts and evaluated against the same benchmark. Secondly, subject to whether the corpus was brought up-to-date w.r.t. the version of ontology used for training (which is not mentioned in any of the publications), the results may not be directly comparable. This is important because concepts may be retired and subsumed under other concepts, with the original ID becoming an alt_id of the new concept, hence leading the corpus to point to virtually non-existing concepts.

These challenges become evident when inspecting the experimental results published by the top three deep learning approaches, listed in Table 1. The table denotes the version of the ontology employed and the other systems used for assessment. Please note that PhenoBCBert does not provide the HPO version information and we hence assumed a version closer to the publication date of the pre-print. Moreover, the authors only report document-level performance metrics. Finally, we included the results we achieved for PhenoTagger in Groza *et al.* (2023) only for illustration purposes because we used a version of HPO different to the one chosen by the PhenoTagger authors.

**Table 1.** Experimental results across all HPO versions available in the literature on the GSC+ corpus.

	2019-11 (PhenoTagger)						2019-09 (PhenoBERT)						2023-07 (PhenoBCBert)			2022-02 (T-BLAT)		
	Mention-level			Document-level			Mention-level			Document-level			Document-level			Document-level		
	P	R	F1	P	R	F1	P	R	F1	P	R	F1	P	R	F1	P	R	F1
OBO Tagger	*0.85*	0.53	0.65	0.8	0.56	0.66							0.81	0.56	0.66			
NCBO							0.72	0.46	0.56	0.66	0.5	0.57	0.77	0.52	0.62	0.66	0.49	0.56
ClinPhen							0.64	0.41	0.5	0.51	0.41	0.45	0.59	0.41	0.48	0.63	0.65	0.64
Doc2HPO	0.79	0.59	0.67	0.76	0.61	0.68	0.77	0.47	0.58	0.71	0.49	0.58	0.75	0.6	0.67	0.8	0.49	0.61
Monarch	0.79	0.61	0.69	0.75	0.6	0.67										*0.82*	0.5	0.62
NeuralCR	0.78	0.58	0.67	0.74	0.6	0.66	0.74	0.66	0.7	0.71	0.67	0.69	0.73	0.61	0.66			
PhenoTagger	0.78	*0.72*	**0.75**	0.77	*0.74*	0.75	0.79	0.63	0.7	0.78	0.68	0.73	0.72	0.76	0.74	0.77	0.67	0.72
PhenoBERT							0.8	0.66	0.72	0.79	0.7	0.74						
PhenoBCBERT													0.74	*0.81*	**0.77**			
FastHPOCR *(corpus not aligned)*	0.71	0.69	0.7	0.74	0.71	0.72	0.71	0.69	0.7	0.74	0.71	0.72	0.74	0.71	0.72	0.74	0.71	0.72
FastHPOCR *(corpus aligned)*	0.8	0.69	0.74	*0.82*	0.71	**0.76**	*0.8*	*0.69*	**0.74**	*0.82*	*0.71*	**0.76**	*0.81*	0.71	0.76	0.81	*0.71*	**0.76**

Bold values denote the best in class F1 score, while italic values denote best in class Precision or Recall.

As it can be observed that no two reports list the exact same values. Indeed, the changes are not necessarily meaningful—i.e. 0.01 to 0.05 (e.g. mention-level-F1 for PhenoTagger reported by PhenoTagger as 0.75 and by PhenoBERT as 0.7). They do, however, become relevant in the context of similar changes in values being reported as surpassing the state-of-the-art: e.g. document-level-F1 for PhenoTagger reported by PhenoTagger as 0.75 and by PhenoBERT as 0.73, while PhenoBERT itself achieving 0.74. Similarly, PhenoBCBert reports document-level-F1 for PhenoTagger as 0.74, while PhenoBCBert itself achieves 0.77. Remarkably, our own experiments are more aligned to PhenoBERT (in particular when considering the document-level-Recall) than to PhenoBCBert.

Given the lack of a uniform and deterministic platform to compute these results, it is impossible to conclude which of these reports have resulted from an experimental setup closer to the original. Unfortunately, this challenge is not specific only to the phenotype domain, but rather spread across all domains that use a fixed corpus and an evolving ontology—e.g. the exposome, social determinants of health, experimental factors, etc.

In order to showcase the impact of both the version of the ontology, as well as the need for aligning the corpus to the latest set of HPO IDs, in Table 1 we list the results achieved by our solution across all HPO version used by the previous approaches and in both alignment settings. When the corpus is aligned to the HPO version (strictly in terms of resolving retired IDs to new ones) FastHPOCR matches or surpasses PhenoTagger and PhenoBERT:

- 0.76 document-level-F1 on 2019-11 versus 0.75 document-level-F1 PhenoTagger
- 0.76 document-level-F1 on 2019-09 versus 0.74 and 0.73 document-level-F1, respectively, for PhenoBERT and PhenoTagger

and fails to match PhenoBCBert 0.76 document-level-F1 on 2023-07 (versus 0.77 document-level-F1 PhenoBCBert), although it does achieve a better precision 0.81 versus 0.74.

As expected, the results differ when alignment is missing, with all performance scores decreasing by 0.03–0.04, hence supporting the argument of including an increased transparency of the experimental setup to foster reproducibility and an appropriate comparison of the results. Table 2 lists a second set of experiments performed on the BIOC-GS corpus we initially documented in Groza *et al.* (2023). Our approach surpasses PhenoTagger—document-level-F1 0.62 versus 0.61—with an increase in recall from 0.52 to 0.57. We note, however, a drop in precision between the two approaches—from 0.74 for PhenoTagger to 0.67 for FastHPOCR. Reasons behind the lower value for precision include the annotation of nested entities—which are not marked in the gold standard (e.g. *hernia* as a nested entity within *umbilical hernia*) and incorrect candidate generation due to the format of the gold standard entries—e.g. in the entry *NOSE: Broad and wide nasal bridge.* FastHPOCR identifies *NOSE Broad* as an invalid candidate. In the context of the application domains mentioned in Section 1, a higher recall is desirable for the clinical domain, since the patient profile creation is almost always accompanied by a review step. A higher precision, however, is beneficial when considering literature annotation given the sheer amount of data being processed and the lack of human feedback on the outcomes.

**Table 2.** Experimental results on the BIO-GS corpus.

	P	R	F1
PhenoTagger	0.74	0.52	0.61
ClinPheno	0.47	**0.57**	0.52
Doc2HPO	**0.84**	0.29	0.43
Monarch	0.47	0.46	0.46
NCBO	0.78	0.41	0.54
FastHPOCR	0.67	**0.57**	**0.62**

Bold values denote the highest score for the corresponding metric.

We make a final note on the efficiency of our solution—from a resource usage perspective—to support the second challenge we have raised in Section 1—i.e. the evolution of ontologies leads to a need for reanalysis of the domain corpora (e.g. clinical notes or publications). Our solution requires ∼3 min to index a new HPO version, ∼5 s to annotate 10 000 abstracts (on average each abstract has ∼1400 characters; excluding I/O operations) and ∼50 s to annotate 100 000 abstracts on an AWS T3.medium machine (2 vCPU, 4GB RAM). Given the linear relationship between time and number of abstracts, a complete reanalysis of PubMed (∼30M abstracts) would take 4 h15 min on an extremely low-end machine and cost ∼$0.208.

## 5 Discussion

### 5.1 Error analysis

Concept recognition methods are usually affected by a rather consistent set of errors, some of which are more prevalent in the case of dictionary-based approaches such as ours. We performed a thorough error analysis against the annotations produced on the aligned GSC+ corpus with HPO version 2019-09/2019-11 and listed below our findings.

Eight types of errors were found:

- Coordinated terms (∼15%)—e.g. “*oral, and ophthalmic anomalies*” (target concept—*Oral anomalies–*HP: 0031816) or “*cleft lip and palate*” (target concept—*Cleft palate–*HP: 0000175);
- Non-contiguous representations (∼6%): “*dysplastic left kidney*” (target concept—*Renal dysplasia–*HP: 0000089);
- Lack of synonyms (∼15%): “*Developmental defects*” (target concept—*Global developmental delay–*HP: 0001263);
- Lack of context (∼28%): “*Laughter*” (target concept—*Inappropriate laughter–*HP: 0000748) or “*lipomas*” (target concept—*Multiple lipomas–*HP: 0001012);
- False positives (∼16%): “*Hypotonia*” (identified as *Generalized hypotonia*) or “*Fell*” (identified as *Falls*);
- Incorrect boundary detection (∼2%): False positives due to consecutive placement of tokens in text;
- Incorrect degree of specificity (∼7%): “*Gingival papules*” (target concept—*Abnormality of the gingiva–*HP: 0000168) or “*ovarian fibrosarcoma*” (target concept—*Abnormality of the ovary–*HP: 0000137);
- Missing annotations (∼11%): True positives identified as false positives because the corpus is lacking the annotations.

We anticipated some of the errors—such as the coordinated terms or the lack of synonyms—as we discuss in the Section 5.2. It is, however, important to note that our boundary detection approach does not lead to a significant number of errors. Most of the other types of errors—in particular those requiring context information—can be addressed via partial matching combined with context disambiguation (although this will most likely also increase the number of false positives).

Finally, we analysed changes in errors across the various versions of the ontology:

- 2019-09 to 2019-11: produced no changes;
- 2019-11 to 2022-02: produced 13 changes:
  - One change in HPO ID (identified as false positive);
  - Five actual false positives due to new terms;
  - Seven correctly identified annotations—mostly because of new synonyms;
  - 2022-02 to 2023-07: produced one change;
  - Missing annotation—HP: 0001756—Vestibular dysfunction.

### 5.2 Limitations

Our dictionary-based solution suffers from the same limitations as all other similar approaches—as also shown in the error analysis. Firstly, it is bound to the tokens we have captured and consolidated and is unable to identify synonyms and terms that have not been defined explicitly in the ontology or *a priori* in an extension of our approach. BERT-based approaches—with pre-computed embeddings on large corpora—have a higher chance of filling this gap. An appropriate study would, however, be required to quantify the impact of these unseen synonyms on the performance metrics.

Secondly, our current matching strategy relies on identifying contiguous spans of text where punctuation and coordination breaks continuity. A complementary matching strategy would be required to enable identification of coordinated terms (e.g. “*short and broad toes*”—leading to *short toes* and *broad toes*) or non-canonical structures (e.g. “*the stature is short*”—leading to *short stature*). The results published by the BERT models showcase a lack of ability to address this challenge. However, GPT-based solutions that combine prompt engineering for named entity recognition with a subsequent entity linking step can provide a successful solution.

Finally, a limitation associated with CR in general is the lack of a context-dependent disambiguation, which is particularly relevant—in the case of phenotypes—for terms that contain common English words—e.g. “*Negativism*” (possibly encountered often as the token “*negative*” in publications) or “*Blindness*” (carrying several context-dependent meanings—i.e. double-blind review versus blind spots versus the patient was blind). Similar to the challenge above a disambiguation module applied as a post-processing step would lead to positive results, including a solution built using LLMs to verify the relevance of the extracted phenotypes from a medical perspective to the original context.

### 5.3 Updated GSC corpus

**Figure 4.**
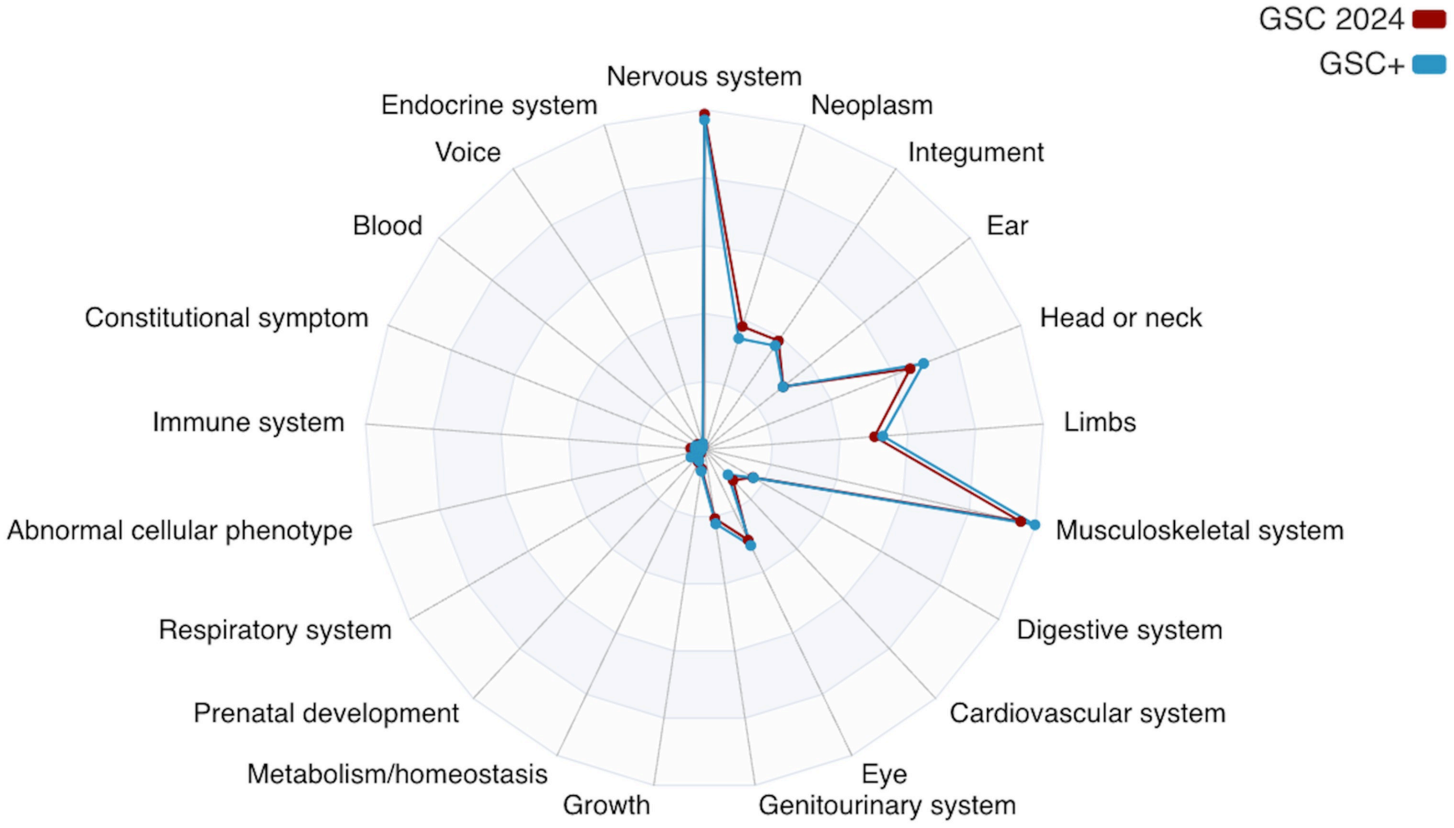
High-level comparison between the GSC+ corpus and the new GSC 2024 corpus, using the top level HPO abnormalities as reference.

Given the significant number of changes in the ontology since the last update of the GSC+ corpus, we proceeded with aligning it to HPO version 2024-02 and made it available at: https://github.com/tudorgroza/code-for-papers/tree/main/gsc-2024. Table 3 lists the differences between the two corpora. Notably, we introduced 137 new annotations and increased the coverage of unique phenotype concepts to 486 from 433. In practice, 21 concepts were removed (via subsumption under other concepts) and 74 new concepts added. The overall profile of the corpus has not changed, as depicted in Fig. 4.

**Table 3.** Comparison between the GSC+ corpus and the new GSC 2024 corpus.

	GSC+	GSC 2024
Total annotations	2773	2910
Total non-pheno annotations	503	503
Total concepts	454	507
Unique non-pheno concepts	21	21
Unique pheno concepts	433	486

A re-run of the evaluation experiments using the 2024-02 version of HPO on the updated corpus has yielded significantly improved results: 0.94 P, 0.78 R, and 0.85 F1 (mention-level), and 0.96, 0.79, 0.87 P, R, and F1, respectively (document-level).

## 6 Conclusion

The phenotype CR field is under active development and will continue to produce novel approaches, underpinned both by the improvements brought to LLMs but also by an increasing use of HPO in conjunction with EHR data. While most of the novel methods will focus on machine learning techniques, in this article we showed that “traditional” dictionary-based approaches can be employed efficiently and at speed to cater for the continuous evolution of HPO and for re-analysis (refresh) of the previously analysed data with new (or updated) concepts.

Our proposal relies on a collection of clusters of morphologically equivalent tokens aimed at addressing lexical variability and on a closed-world assumption applied during CR to find candidates and perform entity linking. The solution produces state-of-the-art results augmented by an incomparable speed—10 000 publication abstracts in 5 s. To address some of the limitations associated with dictionary-based methods in general, we intend to develop a series of post-processing modules to introduce context-disambiguation and partial matching.
